# Docetaxel-Loaded Chitosan-Cholesterol Conjugate-Based Self-Assembled Nanoparticles for Overcoming Multidrug Resistance in Cancer Cells

**DOI:** 10.3390/pharmaceutics12090783

**Published:** 2020-08-19

**Authors:** Chao-Feng Mu, Fude Cui, Yong-Mei Yin, Hyun-Jong Cho, Dae-Duk Kim

**Affiliations:** 1 College of Pharmacy and Research Institute of Pharmaceutical Sciences, Seoul National University, Seoul 08826, Korea; cmu2005@126.com (C.-F.M.); yinyongmei@nankai.edu.cn (Y.-M.Y.); 2 School of Pharmacy, Shenyang Pharmaceutical University, Shenyang 110016, China; syphucuifude@163.com; 3 College of Pharmacy, Nankai University, Tianjin 300071, China; 4 College of Pharmacy, Kangwon National University, Chuncheon, Gangwon 24341, Korea

**Keywords:** chitosan, cholesterol, docetaxel, multidrug resistance-overcoming, nanoparticles

## Abstract

Cholesteryl hemisuccinate (CHS)-conjugated chitosan (CS)-based self-assembled nanoparticles (NPs) were developed for enhancing the intracellular uptake of docetaxel in multidrug resistance (MDR)-acquired cancer cells. CHS-CS was successfully synthesized and self-aggregation, particle size, zeta potential, drug entrapment efficiency, and in vitro drug release of docetaxel-loaded CHS-CS NPs were tested. The optimized NPs had a mean hydrodynamic diameter of 303 nm, positive zeta potential of 21.3 mV, and spherical shape. The in vitro release of docetaxel from the optimized CHS-CS NPs in different pH medium (pH 6.0 and 7.4) revealed that the release was improved in a more acidic condition (pH 6.0), representing a tumor cell’s environment. The superior MDR-overcoming effect of docetaxel-loaded CHS-CS NPs, compared with docetaxel solution, was verified in anti-proliferation and cellular accumulation studies in MDR-acquired KBV20C cells. Thus, CHS-CS NPs could be potentially used for overcoming the MDR effect in anticancer drug delivery.

## 1. Introduction

In the past few decades, nanoparticles (NPs), based on natural or synthetic polymers, have been widely investigated as drug delivery carriers due to their unique characteristics [1,2,3,4,5,6,7]. Biocompatible polymers, such as hyaluronic acid, poly(lactic-co-glycolic acid), and cellulose, have been introduced to design NPs for drug delivery. These NPs have passive targeting by an “enhanced permeability and retention (EPR) effect”, extended circulation time with sustained drug release, and reduction of systemic toxicity [8]. Chitosan (CS), as one of the natural polymers, and its derivatives have been introduced to make NPs for their biocompatibility [9,10]. CS can be prepared from chitin via the deacetylation process. It is one of the polysaccharides and has cationic, basic, and mucoadhesive characteristics [11]. It can be physically and chemically modified and thus it can be used to make various types of nano- and micro-size particles, which can be further applied to drug delivery and tissue engineering [11]. Among CS derivatives, NPs based on amphiphilic CS derivatives have received increasing interest for their simple preparation methods. Moreover, the morphology, hydrophobicity, and particle size of these NPs can be controlled by the molecular weight of CS and the types and the degree of substitution (DS) of hydrophobic groups conjugated with CS [9,12,13]. The hydrophobic microdomains formed by the intra- and/or intermolecular association of hydrophobic segments enable the NPs to act as the reservoir of hydrophobic drugs [14].

However, EPR effect-based tumor targeting strategies have also been challenged by several characteristics of the tumor microenvironment, which include poor cellular uptake, drug efflux, high interstitial fluid pressure, high density of extracellular matrix, and hypoxia [15]. Among them, low membrane permeability and over-expressed efflux transporters, such as P-glycoprotein (P-gp) and multidrug resistance-associated protein (MRP), in multidrug resistance (MDR)-acquired cancer cells have been major obstacles for the passive targeting of NPs in tumor regions [16,17]. Therefore, it is necessary to enhance the permeation of the cellular membrane and to block the efflux mechanism of cancer cells in the treatment of MDR-acquired cancer. It was reported that NPs based on the amphiphilic polymers (including CS derivatives) can overcome MDR in cancer cells [18,19].

In this study, CS was grafted with cholesteryl hemisuccinate (CHS), as a hydrophobic residue. CHS is a type of cholesterol-mimicking detergent and it can be easily applied to fabricate nano-sized drug delivery vehicles (i.e., NP and liposome) [20,21]. CHS was introduced to NPs as a hydrophobic moiety for the reservoir of hydrophobic anticancer drugs and its good biocompatibility. Docetaxel is a second-generation taxane, which is an effective anticancer drug against advanced and metastatic breast cancer, non-small cell lung cancer, and advanced gastric cancer [22]. In spite of its broad application to several types of malignant tumors, its poor water solubility does not meet the clinical dose without the aid of solubilizers. For intravenous injection, several formulations have been developed to improve the solubility and tumor targeting efficiency [9,10,11,12,13,14]. In addition, side effects, such as hypersensitivity reactions, fluid retention, and peripheral neurotoxicity [23], caused by its currently available commercial formulation have considerably overshadowed its clinical use. In this study, docetaxel was loaded to the hydrophobic inner cavity of CHS-CS NPs for its delivery. Although cholesterol-modified CS NPs were previously reported [24,25], their application for overcoming MDR in cancer cells has not been fully investigated. To the best of our knowledge, this may be the first report regarding the MDR-overcoming strategy of docetaxel-loaded CHS-CS NPs in cancer therapy. It is expected that MDR circumventing effects of CHS-CS NPs may amplify the anticancer activities of entrapped drugs. The objective of this study was to investigate the feasibility of self-assembled NPs of the CHS-CS conjugate for enhancing intracellular accumulation and inhibiting drug efflux in MDR-acquired cancer cells.

## 2. Materials and Methods

### 2.1. Materials

Docetaxel was purchased from Taihua Co. (Xi’an, China). CS (average MW: 34 kDa, deacetylation: 97%) was supplied by Kitto Life Co., Ltd. (Pyeongtaek, Korea). CHS, *N*-(3-dimethylamino propyl)-*N*′-ethylcarbodiimide hydrochloride (EDC), *N*-hydroxyl succinimide (NHS), dimethylformamide (DMF), 2,4,6-trinitrobenzenusulfonic acid (TNBS), and pyrene were purchased from Sigma-Aldrich Inc. (St. Louis, MO, USA). RPMI-1640 media, penicillin-streptomycin, and fetal bovine serum (FBS) were purchased from Life Technologies Corp. (Carlsbad, CA, USA). All other chemicals were reagent grade and obtained from commercial sources.

### 2.2. Cell Culture

KB (human carcinoma) cell was purchased from the American Type Culture Collection (Rockville, MD, USA). KBV20C (MDR-acquired KB) cells were kindly gifted by Prof. Dong Kwon Rhee (College of Pharmacy, Sungkyunkwan University, Suwon, Korea). KB and KBV20C cells were cultured in RPMI 1640 media supplemented with 10% FBS, 2 mM L-glutamine, 10 mM HEPES, 24 mM NaHCO_3_, and 1% penicillin/streptomycin.

### 2.3. Synthesis of Cholesteryl Hemisuccinate-Conjugated Chitosan (CHS-CS) and Its Verification

CS was coupled with CHS via an amide bond as reported with slight modification [24]. Briefly, CS (15 mg), EDC (4 mg), and NHS (2 mg) were dissolved in 0.4 mL of distilled water (DW), followed by adding dropwise into 4 mL of DMF/DW mixture (9:1, *v/v*) under stirring at 500 rpm at room temperature. Then, an aliquot of DMF solution containing CHS (1.0 mg/mL) was added dropwise to the mixture for 4 h. The reaction was conducted for 48 h, then the reaction mixture was poured into 200 mL methanol/ammonia solution (7/3, *v/v*). The precipitates were filtered, washed thoroughly with methanol and DW, and then dialyzed against DW for 48 h to remove water-soluble by-products. The resultant CHS-CS was freeze-dried. The DS (the number of CHS groups per 100 glucosamine units of CS) was controlled by varying the amount of CHS, determined by the TNBS method [23]. The formation of the CHS-CS conjugate was confirmed by proton nuclear magnetic resonance (^1^H-NMR) (Avance DRX-400, Bruker, Bremen, Germany) analysis.

### 2.4. Self-Aggregation Behavior of CHS-CS

The self-aggregation behavior of CHS-CS was tested with the fluorescence probe technique using pyrene [26]. CHS-CS dispersion specimens were prepared and pyrene was added at 6 × 10^−7^ M. They were left in the dark for 24 h to reach equilibrium at room temperature. Fluorescence spectra of pyrene were obtained by using a fluorescence spectrometer (FP-6500, Jasco Co., Tokyo, Japan) at an excitation wavelength of 330 nm. The emission spectra were recorded from 360 to 460 nm. The peak height intensity ratio (*I_397_/I_373_*) was plotted against the concentration of CHS-CS. The critical aggregation concentration (CAC) value was taken from the inflection point of the curve.

Self-aggregates of blank CHS-CS NPs were prepared by dispersing them in the aqueous medium. The particle size of blank NPs was measured using a NICOMP 370 Submicron Particle Sizer (Particle Sizing Systems, Santa Barbara, CA, USA). The zeta potential of CHS-CS NPs in DW was measured by an electrophoretic light scattering (ELS) spectrophotometer (ELS-8000, Otsuka Electronics Co. Ltd., Osaka, Japan).

### 2.5. Preparation and Particle Characterization Tests of CHS-CS Nanoparticles (NPs)

Docetaxel-loaded CHS-CS NPs were prepared by two different methods [27,28]. In preparation method I, CHS-CS (4 mg) was dispersed in 2 mL of 0.01% (*v/v*) acetic acid solution under stirring (200 rpm) at room temperature and it was mixed using a probe sonicator for 5 min. Then, 0.2 mL of acetone containing docetaxel (0.4, 0.6 or 1.0 mg/mL) was added dropwise to the CHS-CS dispersion and it was sonicated for 10 min. The resulting mixture was stirred (200 rpm) for 8 h at room temperature and filtered through a syringe filter (0.8 μm of pore size). Then, it was dialyzed with a dialysis membrane (molecular weight cut-off (MWCO): 3.5 kDa) against DW for 12 h and lyophilized. In preparation method II, docetaxel (0.08, 0.12, or 0.2 mg) and CHS-CS (4 mg) were added into 2 mL of DW/dimethyl sulfoxide (DMSO) (2:8, *v/v*) mixture and stirred (200 rpm) for 3 h at room temperature. Then, it was dialyzed in DW for 12 h using a dialysis membrane (MWCO: 3.5 kDa). The dialyzed preparations were then filtered using a syringe filter (pore size: 0.8 μm) and lyophilized.

Particle size and zeta potential of drug-loaded CHS-CS NPs dispersed in DW were measured using a NICOMP 370 Submicron Particle Sizer (Particle Sizing Systems) and ELS spectrophotometer (ELS-8000, Otsuka Electronics Co. Ltd., Osaka, Japan), respectively. The morphology of NPs was observed by transmission electron microscopy (TEM; Libra 120, Carl Zeiss, Oberkochen, Germany) using phosphotungstic acid solution (2%).

Drug entrapment efficiency and loading content in NPs were determined by high-performance liquid chromatography (HPLC) analysis after the disruption of lyophilized NPs with acetonitrile. Docetaxel was quantitatively analyzed by the HPLC method [29]. The HPLC system consisted of a dual pump (Waters 515, Waters Corp., Milford, CA, USA), auto-sampler (Waters 717 plus), Capcell Pak C18 MG column (4.6 × 250 mm, 5 μm, Shiseido, Tokyo, Japan), and UV detector (Waters 2487) set at 230 nm. Mobile phase was a mixture of acetonitrile and water (65:35, *v/v*), filtered through a 0.45-μm syringe filter, and eluted at a flow rate of 1.0 mL/min. Docetaxel concentrations were determined using 20 μL of injection volume at room temperature. The inter- and intra-day variance of this HPLC method was within the acceptable range. Lower limit of quantification value was 100 ng/mL in this analytical method.

### 2.6. In Vitro Release Test of Docetaxel

In vitro release profile of docetaxel from NPs was evaluated according to the reported method with slight modification [30]. An aliquot (0.2 mL) of docetaxel-loaded NPs (2%, *w/v*) dispersion was placed in a dialysis tube (MWCO: 6–8 kDa, Gene Bio-Application Ltd., Yavne, Israel) and transferred to 20 mL of phosphate buffer saline (PBS, pH 6.0 and 7.4) as a release medium, which is maintained at 37.0 ± 0.5 °C with a stirring rate of 100 rpm. An aliquot of the release media (0.2 mL) was sampled at predetermined time intervals (0, 1, 2, 4, 6, 8, 12, 24, 36, and 48 h) and replaced with the equal volume of fresh medium. Docetaxel in aliquots was quantitatively analyzed by the HPLC system described above. The released amount (%) of drug was calculated by the following formula.

(1) $$\mathrm{Released}\;\mathrm{amount}\;\mathrm{of}\;\mathrm{drug}\;\left(\%\right)=\;\frac{(C_{t}\times V_{R})+\left(\sum_{n=1}^{t-1}C_{n}\times V_{S}\right)}{Input\;amount\;of\;drug}\times 100$$

where *C_t_*, *V_R_*, and *V_s_* indicate drug concentration in the release medium at the corresponding time point, volume of release medium, and sampling volume, respectively.

### 2.7. In Vitro Cytotoxicity Assay

In vitro cytotoxicity of docetaxel-loaded CHS-CS NPs was determined in KB and KBV20C cells by colorimetric assay. Cells were seeded in a 96-well plate at a density of 5 × 10^3^ cells per well and incubated for 24 h at 37 °C in a humidified atmosphere with 5% CO_2_. Cells were then incubated for 48 h with docetaxel-loaded CHS-CS NPs (1, 5, and 10 ng/mL of docetaxel for KB cells and 10, 50, and 100 ng/mL of docetaxel for KBV20C cells), blank CHS-CS NPs, and docetaxel solution diluted with the cell culture medium. Then, cells were rinsed with PBS and 3-(4,5-dimethylthiazol-2-yl)-2,5-diphenyltetrazolium bromide (MTT) solution was added to the cells. The non-reduced MTT and media were discarded after 3 h of incubation period. Then, each well was washed with PBS, after which DMSO was added to dissolve the residual precipitates. The absorbance was read at 560 nm using a microplate reader (Molecular Devices Corp., Sunnyvale, CA, USA).

### 2.8. Cellular Uptake Study

For the cellular uptake study, KB and KBV20C cells were seeded in 24-well plates at a density of 1 × 10^5^ cells/well and incubated for 24 h. Then, the cell culture medium was replaced with fresh culture medium containing docetaxel-loaded CHS-CS NPs or free docetaxel and incubated for 4 or 8 h at 37 °C. Cells were then washed twice with cold PBS and incubated with 0.1 mL of 2% (*w/v*) sodium dodecyl sulfate (SDS) solution for 30 min at 37 °C. Protein content in cell lysate was determined by Pierce^TM^ BCA protein assay kit (Thermo Fisher Scientific, Waltham, MA, USA), while docetaxel was extracted from the cell lysate with equal volume of acetonitrile. The mixture of cell lysate and acetonitrile was centrifuged at 13,000 rpm for 5 min and the supernatant was used for HPLC analysis.

### 2.9. Statistical Analysis of Data

All the experiments in the study were performed at least three times and the data were expressed as the mean ± standard deviation (S.D.). A two-tailed unpaired student’s *t*-test was performed for statistical analysis.

## 3. Results and Discussion

### 3.1. Synthesis of CSH-CS and Its Self-Assembly Properties

**Figure 1:**
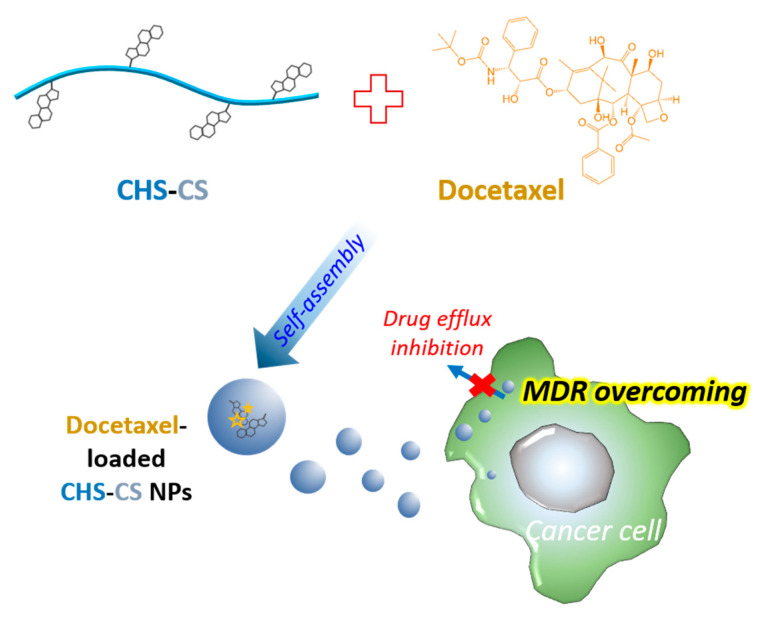
Schematic illustration of cholesteryl hemisuccinate-conjugated chitosan (CHS-CS) nanoparticles (NPs) and multidrug resistance (MDR)-overcoming in cancer cells.

**Figure 2:**
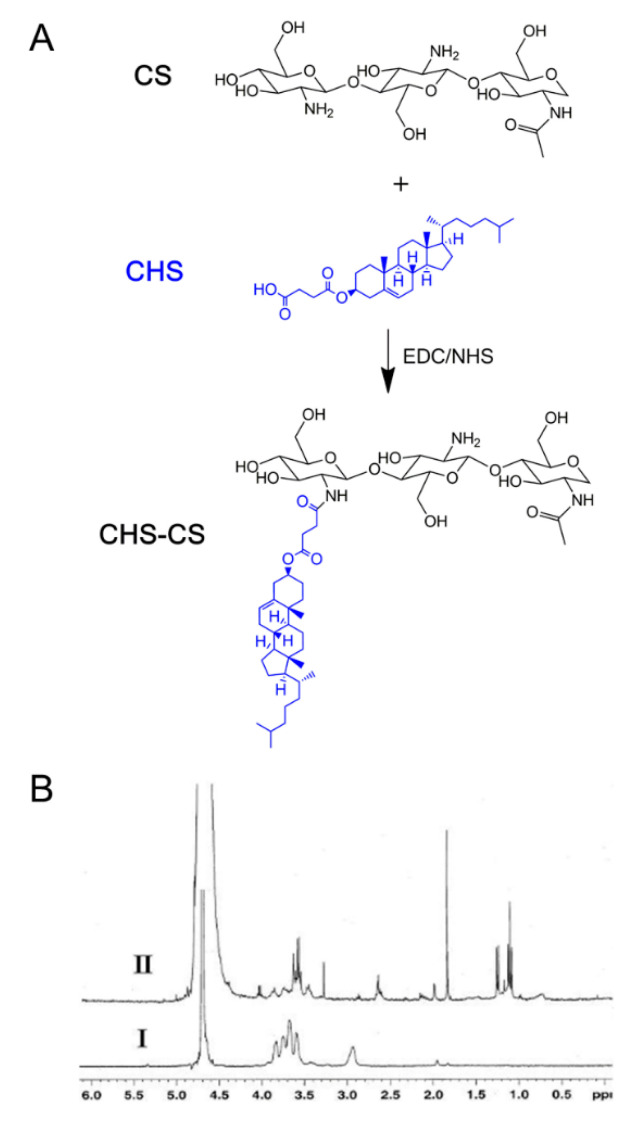
Synthesis and identification of CHS-CS. (**A**) Synthetic sketch of CHS-CS. (**B**) Proton nuclear magnetic resonance (^1^H-NMR) spectra of chitosan (CS) (I) and CHS-CS (II).

**Figure 3:**
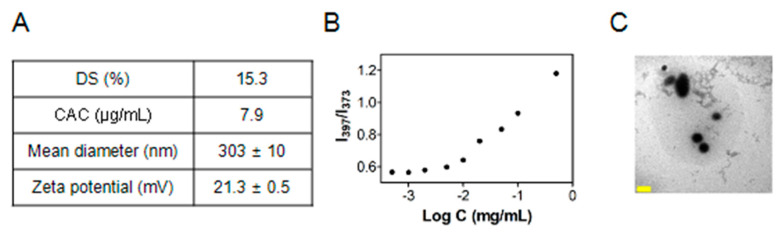
Particle properties of CHS-CS NPs. (**A**) Particle characterization of CHS-CS NPs. (**B**) Critical aggregation concentration (CAC) determination of CHS-CS. *I_397_/I_373_* values according to log*C* are plotted. (**C**) Transmission electron microscopy (TEM) image of CHS-CS NPs. The length of the yellow bar included in the image is 200 nm.

Amphiphilic CS derivatives were synthesized to fabricate self-assembly nanostructures in the aqueous environment, thereby overcoming MDR in cancer cells and selectively delivering the anticancer drug to the cancer cells (Figure 1). As shown in Figure 2A, CHS-CS was synthesized by an amide bond formation between the carboxylic acid group of CHS and the amino group of CS, with the aid of EDC and NHS. The conjugation of CHS to CS was confirmed by ^1^H-NMR analysis, showing the characteristic shifts of CHS at 0.6–3.0 ppm (Figure 2B). The proton assignment of CS (I) could be as follows (ppm): 1.96 (CH_3_, acetamido group), 2.93 (CH, glucosamine ring), and 3.5–4.0 (CH, glucosamine ring). In the spectrum of CHS-CS (II), the signals at 1.18–1.24 and 1.8 ppm could be assigned to the high-field signal of CHS, while that at 2.62 ppm to methylene protons of succinyl groups, confirming the successful introduction of CHS into CS [15]. The DS of CHS to CS calculated from TNBS data was 15.3% (Figure 3A).

The aggregation behavior of CHS-CS in the aqueous solution was investigated by the steady-state fluorescence probe study, where pyrene was chosen as a fluorescence probe (Figure 3B) [15]. The CAC value usually represents the threshold concentration of a self-aggregate formation by intra- or intermolecular association. The intensity ratio *I_397_/I_373_* from emission spectra was plotted as a function of CHS-CS concentration (Figure 3B). The CAC of CHS-CS calculated from the inflection point was about 7.9 μg/mL (Figure 3A). It was much lower than the reported aggregates with low DS (CAC: 0.02 mg/mL), implying the higher stability of CHS-CS self-aggregates following dilution [24].

Blank CHS-CS NPs prepared by sonication in aqueous medium formed monodisperse NPs with a mean diameter of 303 nm and surface charge of 21.3 mV (Figure 3A). The TEM image demonstrated that CHS-CS self-aggregates were roughly spherical (Figure 3C). However, the mean diameter of these NPs was approximately 200 nm, which was smaller than the value determined by dynamic light scattering (DLS). This may be due to the dehydration (drying) process for the TEM observation, while the particle size of the hydrated state is used to be measured in DLS analysis. Therefore, the particle size determined by DLS was a hydrodynamic diameter and it can be larger than the value read from the TEM image due to the solvent effect [31]. Moreover, the surface charge is a critical parameter on the stability of suspensions and adhesion of particulate systems onto biological surfaces. The positive surface charge (21.3 mV) of CHS-CS NPs due to the amine group of the CS backbone would render higher electrostatic interactions with a negatively charged cellular membrane, resulting in higher cellular entry efficiency [32].

### 3.2. Particle Characterizations of Drug-Loaded NPs

**Figure 4:**
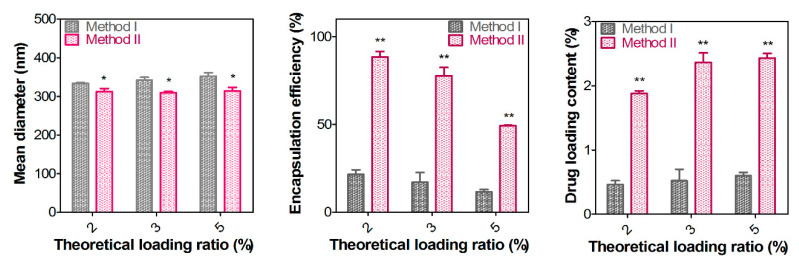
Particle properties of docetaxel-loaded CHS-CS NPs. Mean diameter, encapsulation efficiency, and drug loading content values according to the theoretical loading ratios (2, 3, and 5%) of method I and II are plotted. Encapsulation efficiency (%) = (actual amount of docetaxel in NPs/input amount of docetaxel in NPs) × 100. Drug loading content (%) = (actual amount of docetaxel in NPs/amount of docetaxel-loaded NPs) × 100. * *p* < 0.05, ** *p* < 0.01.

Figure 4 shows the particle properties of drug-loaded CHS-CS NPs prepared with two different methods. Method I and II indicate solvent evaporation and dialysis methods [27,28], respectively, which have been widely used in the fabrication of polymeric NPs. In method I (solvent evaporation method), a poorly water-soluble drug, docetaxel, was dissolved in acetone and it was added to acetic acid solution including CHS-CS and the organic phase (acetone) was eliminated by evaporation during the stirring step. On the contrary, in method II (dialysis method), docetaxel and the CHS-CS solubilized DW/DMSO mixture were put into the dialysis bag and NPs were formed during the dialysis process. The preparation method of NPs may govern the physicochemical properties of fabricated NPs. The increase in the theoretical loading ratio (from 2 to 5%) did not dramatically increase the mean diameter of NPs in both preparation methods. However, the mean diameter of drug-loaded NPs prepared by method II was significantly smaller than that of method I. The different solvent compositions and preparation processes between method I and II seem to influence the particle size. When the incorporation method was changed from method I to method II, the encapsulation efficiency and drug loading content significantly increased from 11–21% to 49–88% and from 0.46–0.60% to 1.88–2.43%, respectively. The solubilization of both the drug and CHS-CS in DW/DMSO mixture (in method II) might contribute to the higher drug encapsulation in NPs. Notably, although the drug loading content increased as the drug-loading increased from 2% to 5% in method II, the encapsulation efficiency significantly decreased from 88.4% to 49.3%. Therefore, a 2% theoretical drug loading ratio and method II were adopted for further studies. In case of method II rather than method I, a smaller amount of CHS-CS is necessary to make NPs including an equivalent amount of docetaxel and it may reduce the toxicities which could be induced by blank CHS-CS NPs. In addition, method II will be more favorable than method I in the scale-up process, economically and environmentally.

**Figure 5:**
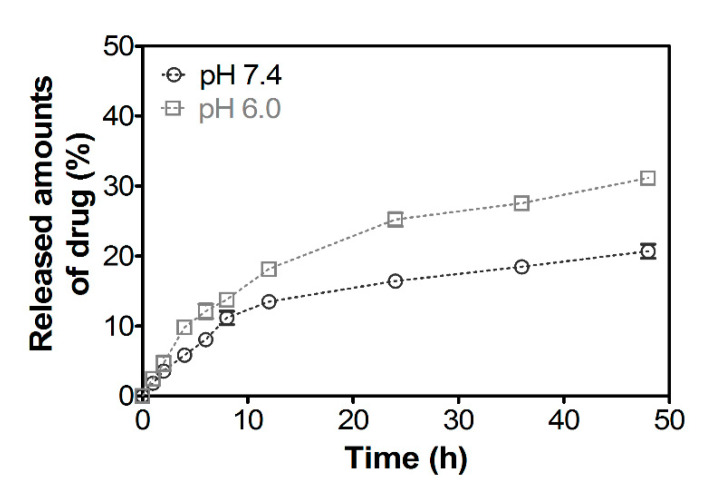
In vitro release profiles of docetaxel from CHS-CS NPs at pH 6.0 and 7.4. Each point represents mean ± S.D. (*n* = 3).

In vitro release study of docetaxel from CHS-CS NPs was carried out in PBS (pH 6.0 and 7.4) at 37 °C, by using a dialysis tube (Figure 5). Docetaxel was released from CHS-CS NPs in a biphasic pattern, which was characterized by an initial rapid release period followed by a step of slower release. The initial burst effect was observed for 12 h, in which nearly 13% and 18% of docetaxel was released from NPs at pH 7.4 and 6.0, respectively. After this initial release period, docetaxel was released in a continuous manner for up to 48 h, and the percentage of cumulative release reached nearly to 20% and 31% at pH 7.4 and 6.0, respectively. The slow release profile of CHS-CS NPs could be due to a rigid hydrophobic domain. Swelling of CHS-CS NPs in a weak acidic condition by protonation of unsubstituted free amino groups in CS can elevate the release rate of docetaxel at pH 6.0 rather than pH 7.4 [33]. It is expected that improved drug release at an acidic pH could contribute to the enhanced drug release following endocytosis of drug-loaded NPs in cancer cells.

### 3.3. Anti-Proliferation Potential

**Figure 6:**
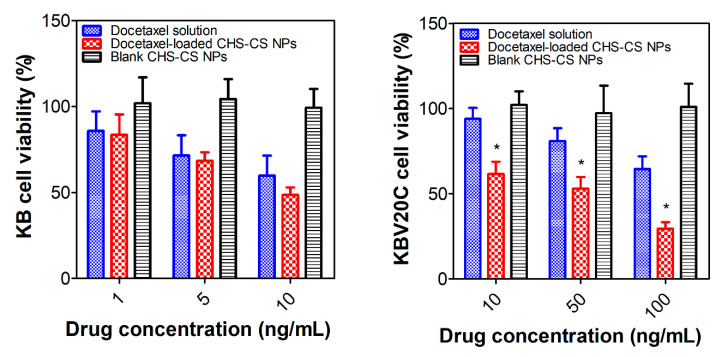
Anti-proliferation assay of designed NPs in KB and KBV20C cells. Docetaxel concentration-dependent cell viability values of docetaxel solution, docetaxel-loaded CHS-CS NPs, and blank CHS-CS NPs in KB and KBV20C cells. Each point represents mean ± S.D. (*n* = 3). * *p* < 0.05.

In vitro cytotoxicity of docetaxel solution, blank CHS-CS NPs, and docetaxel-loaded CHS-CS NPs was investigated in KB and KBV20C cell lines. These cell lines were previously used in studying the anticancer activity and reversal of MDR [34]. Cytotoxicity data of the tested formulations incubated for 48 h in KB and KBV20C cells are shown in Figure 6. In KB cells, the cell viability decreased in a dose-dependent manner (at 1, 5, and 10 ng/mL of docetaxel), but showing no significant difference between the docetaxel solution group and the docetaxel-loaded CHS-CS NPs group. However, in KBV20C cells, the docetaxel-loaded CHS-CS NPs group showed significantly higher cytotoxicity (at 10, 50, and 100 ng/mL of docetaxel) than the docetaxel solution group (*p* < 0.05). These results imply that docetaxel-loaded CHS-CS NPs have MDR-overcoming effects, which might be associated with the enhanced cellular uptake of NPs. It is also interesting to note that the cell viability of blank CHS-CS NPs was almost 100%, indicating that the cytotoxicity is attributed to docetaxel included in the NPs, not NPs themselves.

### 3.4. Cellular Accumulation

**Figure 7:**
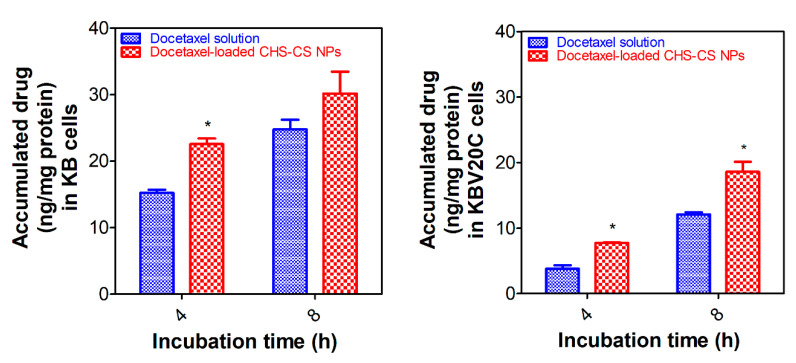
Cellular accumulation assays of designed NPs in KB and KBV20C cells. Incubation time-dependent cellular accumulated amounts of docetaxel in docetaxel solution and docetaxel-loaded CHS-CS NPs-treated groups in KB and KBV20C cells. Each point represents mean ± S.D. (*n* = 3). * *p* < 0.05.

Figure 7 shows the cellular uptake amount of docetaxel after 4 or 8 h of applying docetaxel solution or docetaxel-loaded CHS-CS NPs to KB and KBV20C cells. The cellular uptake amounts in KBV20C cells were lower compared with those in KB cells due to the efflux transporters. These results were consistent with the cell viability study, in which a higher docetaxel concentration was required for KBV20C cells to induce cytotoxic effects (Figure 7). However, in both cell lines, the accumulated amounts of docetaxel after applying drug-loaded CHS-CS NPs were always higher than those of the drug solution group, suggesting that NPs are more efficient than the drug solution in the endocytosis process. It is also notable that the cellular uptake of docetaxel after applying drug-loaded CHS-CS NPs was more significantly enhanced in KBV20C cells rather than KB cells. These results indicate that CHS-CS NPs facilitated their adhesion onto the cell membrane and the subsequent association into the cells, thereby contributing to overcome the MDR effect in cancer cells. Physicochemical properties of polymeric NPs (i.e., particle size, property of materials, surface charge, and ligand conjugation) have significant influences on the extent and mechanisms of their cellular internalization. It was reported that cellular internalization is initiated by nonspecific interactions between NPs and the cell membrane. NPs with a relatively high positive surface charge could adhere strongly to the cell membrane with negative charge by nonspecific electrostatic interactions, leading to the acceleration of the cellular uptake of NPs [35]. CS, which has a positive zeta potential, can interact with the cell membrane by electrostatic interactions. Moreover, surface properties of NPs also could significantly affect the internalization process. It has been reported that cholesterol can modulate the permeability of membranes mainly by reducing the membrane fluidity and membrane thickness, thereby influencing the function of membrane proteins in MDR-acquired cells [36]. In this investigation, although the role of cholesterol was not thoroughly identified, it can be claimed that the CHS-CS NPs, which have the positive charge of CS and the cholesterol-mediated effect on the plasma membrane, could enhance the permeability and the cellular accumulation of anticancer agents in MDR-acquired cancer cells.

## 4. Conclusions

An amphiphilic CHS-CS conjugate was successfully synthesized and its synthesis was confirmed by ^1^H-NMR analysis. The self-aggregation property of the CHS-CS conjugate was identified by fluorescence spectrum, TEM, and DLS studies. Self-assembled CHS-CS NPs bearing hydrophobic inner cavities, in which docetaxel can be entrapped in the NPs, were also successfully prepared. The results of the in vitro cytotoxicity and cellular uptake studies indicated that docetaxel-loaded CHS-CS NPs could be efficiently internalized into the MDR-acquired cancer cells and overcome MDR. These results suggested that CHS-CS NPs can be one of the promising nanocarrier candidates in the treatment of MDR-acquired cancers.
